# Supplementary material for: Aerogels Part 1: A Focus on the Most Patented Ultralight, Highly Porous Inorganic Networks and the Plethora of Their Advanced Applications
Source: Gels. 2025 Sep 8;11(9):718. doi: 10.3390/gels11090718 (PMC12469404; doi:10.3390/gels11090718)
Supplement: Supplementary file 1 [file gels-11-00718-s001.zip › gels-3821978-supplementary.pdf]

gels

Supplementary Materials

Aerogels Part 1. A Focus on the Most Patented Ultralight, Highly Porous Inorganic Networks and the Plethora of Their Advanced Applications

Silvana Alfei

Department of Pharmacy (DIFAR), University of Genoa, Viale Cembrano, 4, 16148 Genoa, Italy
Correspondence: alfei@difar.unige.it; Tel.: +39 010 355 2296

1. Figures

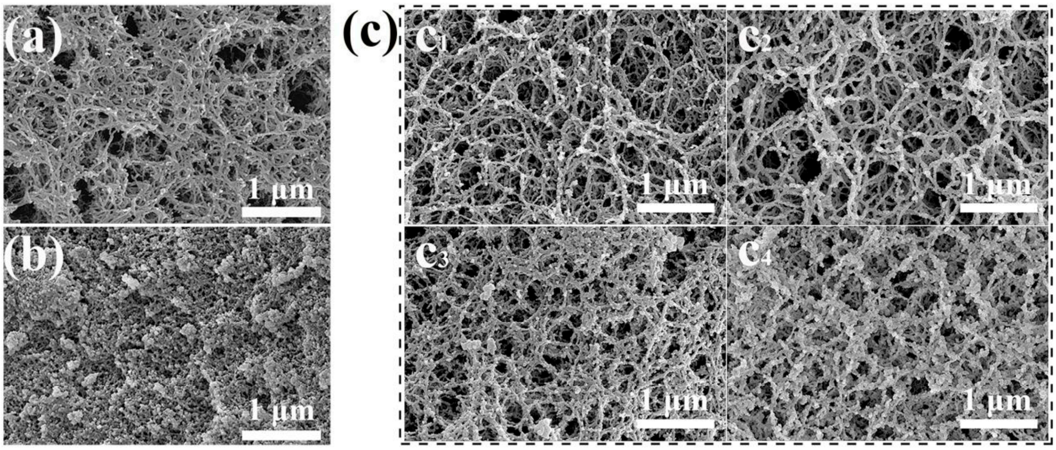

Figure S1. Microstructures of agarose AGs (AA-2), silica AGs (SA-4), and composite AGs (CAs) by scanning electron microscopy (SEM) images, prepared by in situ sol–gel method. This image has been reproduced by an open access article by Yang et al. [557] distributed under the terms and conditions of the Creative Commons Attribution (CC BY) license (https://creativecommons.org/licenses/by/4.0/, accessed on 24 August 2025).

 https://doi.org/10.3390/gels11090718

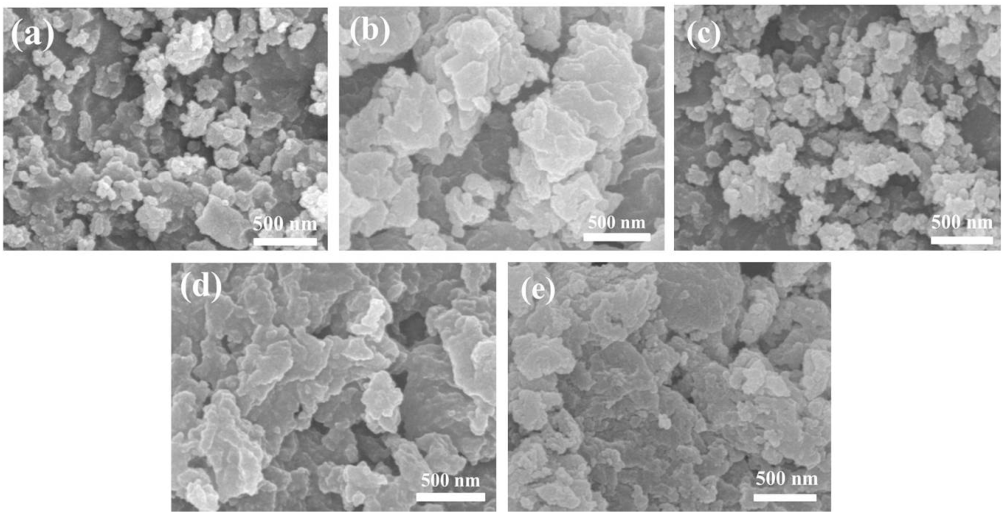

Figure S2. FESEM images of Zr–Mg mixed oxide AGs with Zr/Mg molar ratio of (a) 10/0, (b) 9/1, (c) 8/2, (d) 7/3, and (e) 6/4, prepared by the epoxide addition method. This image has been reproduced by an open access article by Lin et al. [558] distributed under the terms and conditions of the Creative Commons Attribution (CC BY) license (https://creativecommons.org/licenses/by/4.0/, accessed on 24 August 2025).

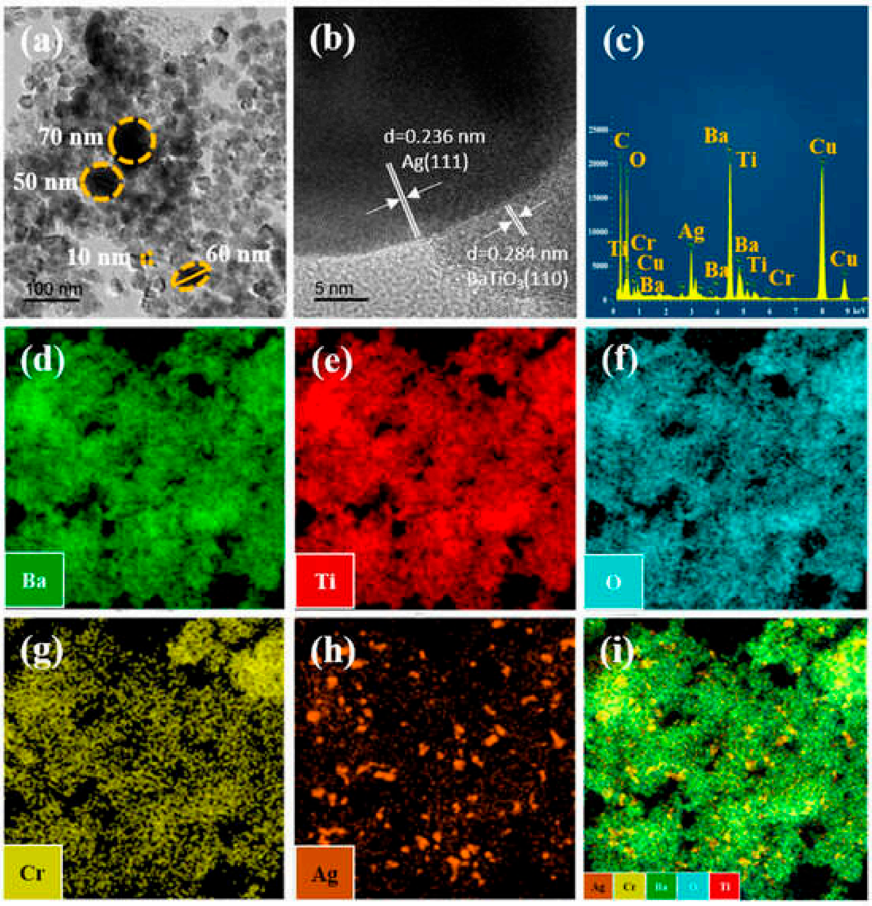

Figure S3. (a) TEM micrographs, (b) HRTEM micrographs, (c) STEM-EDX images, and (d–i) mapping images of Ag-modified Cr-doped $BaTiO_3$ (5% Ag/BTO-Cr010) AGs prepared by using a co-gelation technique that involves two metallic alkoxides and a supercritical drying method, followed by Ag nanoparticles deposition. This image has been reproduced by an open access article by Wu et al. [559] distributed under the terms and conditions of the Creative Commons Attribution (CC BY) license (https://creativecommons.org/licenses/by/4.0/, accessed on 24 August 2025).

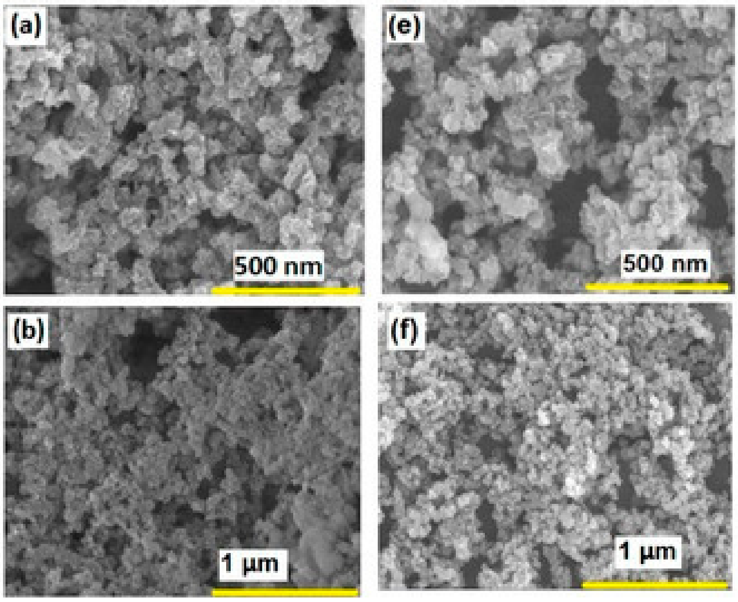

Figure S4. FESEM images of α-Ni(OH)$_2$ AGs prepared by using a two-step sol–gel method followed by a freeze-drying technique (a,b) and of the NiO/Ni aerogels obtained by the prepared α-Ni(OH)$_2$ aerogels through annelation at 400 °C (e,f). This image has been reproduced by an open access article by Ramkumar et al. [560] distributed under the terms and conditions of the Creative Commons Attribution (CC BY) license (https://creativecommons.org/licenses/by/4.0/, accessed on 24 August 2025).

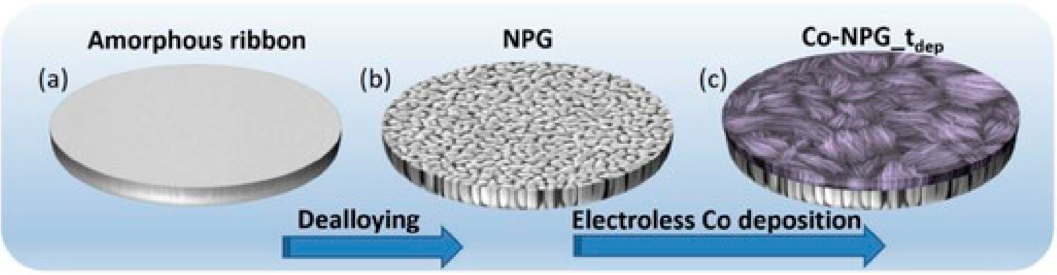

(A)

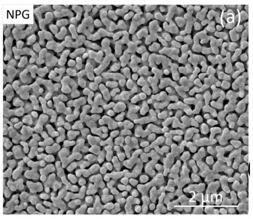

(B)

Figure S5. (A) Schematic preparation of cobalt porous gold nanoparticles (Co-NPG_t$_{dep}$): (**a**) synthesis of the amorphous ribbon through melt spinning process; (**b**) dealloying process to obtain NPG; (**c**) electroless Co deposition onto NPG at selected times. (B) (**a**) SE-SEM image of the porous NPG obtained by dealloying of the amorphous precursor. This image has been reproduced by an open access article by Barrera et al. [561] distributed under the terms and conditions of the Creative Commons Attribution (CC BY) license (https://creativecommons.org/licenses/by/4.0/, accessed on 24 August 2025).

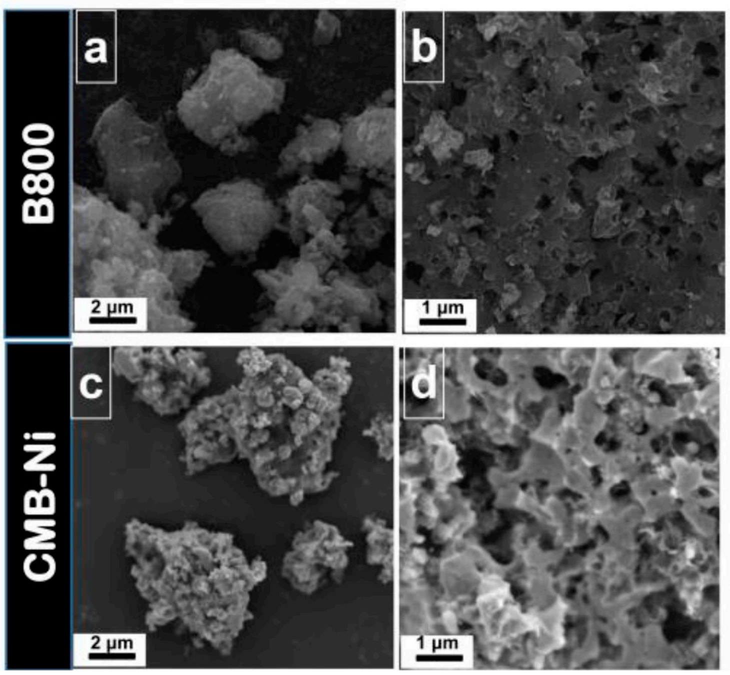

Figure S6. SEM images of biogenetic MnO/C (CMB), namely B800 (Figure S6a, b) and MnO/C/NiO (CMB-Ni) (Figure S6c, d) porous composites. They were obtained for combustion in a tube furnace at 800 °C (4 h, ramp of 5 °C min−1 under an Ar atmosphere) of the MnO_2/bacteria (BMB) and of the MnO_2/bacteria/Ni (BMB-Ni) porous composites prepared by a bio-templated method based on *Pseudomonas putida* cell-surface display technology. This image has been reproduced by an open access article by Liu et al. [562] distributed under the terms and conditions of the Creative Commons Attribution (CC BY) license (https://creativecommons.org/licenses/by/4.0/, accessed on 25 August 2025).

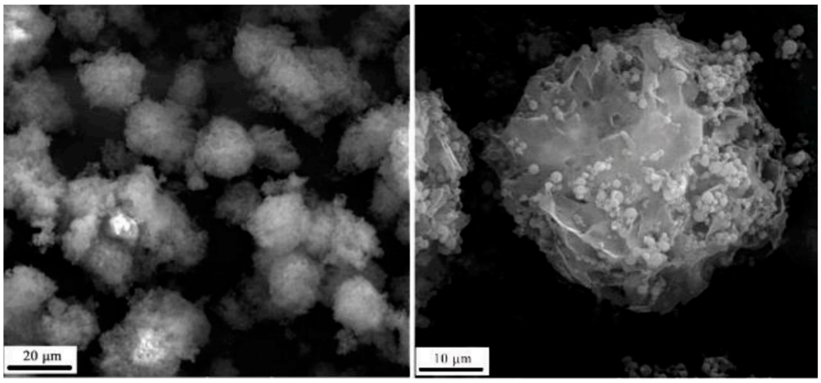

Figure S7. SEM images of biogenic MnO_2/bacteria (BMB) porous composites prepared by a bio-templated method based on *Pseudomonas putida* cell-surface display technology. This image has been reproduced by an open access article by Liu et al. [562] distributed under the terms and conditions of the Creative Commons Attribution (CC BY) license (https://creativecommons.org/licenses/by/4.0/, accessed on 25 August 2025).

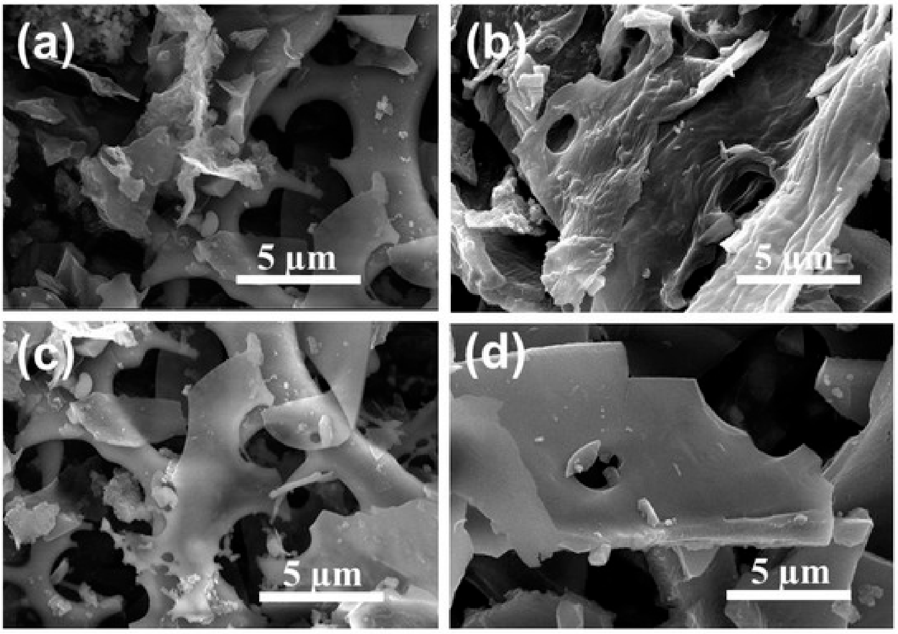

Figure S8. SEM images of biomass-derived porous carbon materials with a good balance between high specific surface area and mesopore volume prepared via a molten chloride salt templating technique and successive KOH activation (MHPC-700, MHPC-800, and MHPC-900) (a,b,c). SEM image of the carbon sample NHPC-700 pre-carbonized in nitrogen atmosphere without molten salt (d). This image has been reproduced by an open access article by Wang et al. [563] distributed under the terms and conditions of the Creative Commons Attribution (CC BY) license (https://creativecommons.org/licenses/by/4.0/, accessed on 25 August 2025).

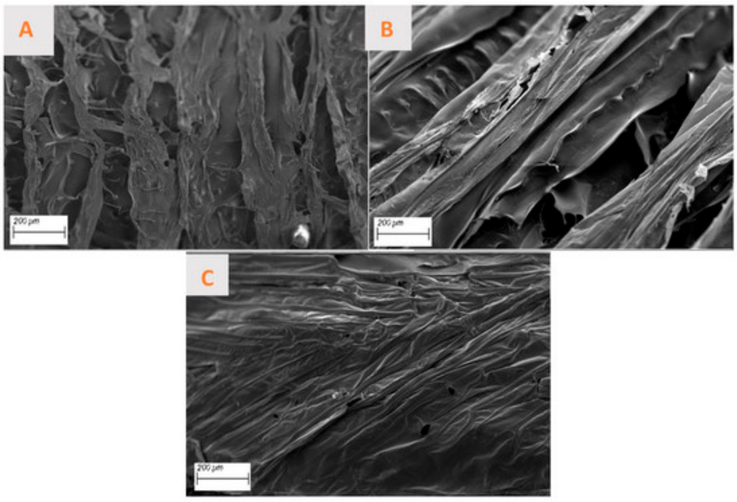

Figure S9. SEM micrographs of hybrid AGs prepared by combining collagen (C) and chitosan (CH) prepared without using nanoparticles (a, reference AG) and using chemical (b) and green (c) iron oxide nanoparticles dispersions, previously synthesized, as building blocks. This image has been reproduced by an open access article by Granados-Carrera et al. [564] distributed under the terms and conditions of the Creative Commons Attribution (CC BY) license (https://creativecommons.org/licenses/by/4.0/, accessed on 25 August 2025).

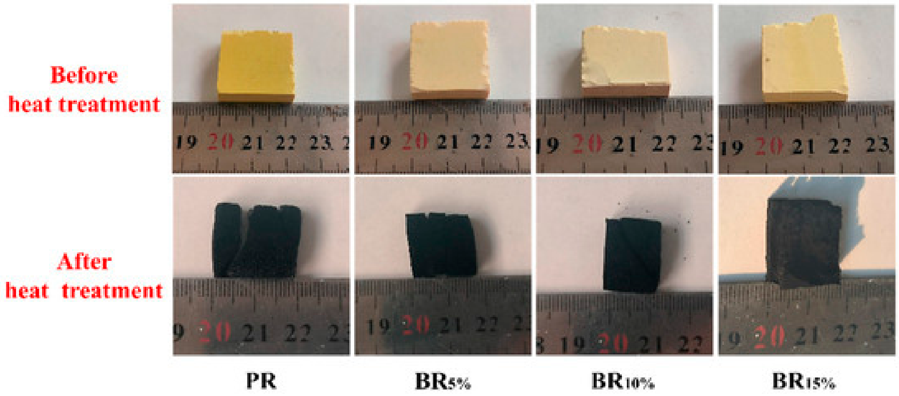

Figure S10. Photos of phenolic resin AGs (PR) and boron-modified phenolic resin AGs containing 5 (BR5%), 10 (BR10%) and 20% (BR20%) boron, before and after high-temperature heat treatment. This image has been reproduced by an open access article by Wu et al. [565] distributed under the terms and conditions of the Creative Commons Attribution (CC BY) license (https://creativecommons.org/licenses/by/4.0/, accessed on 25 August 2025).

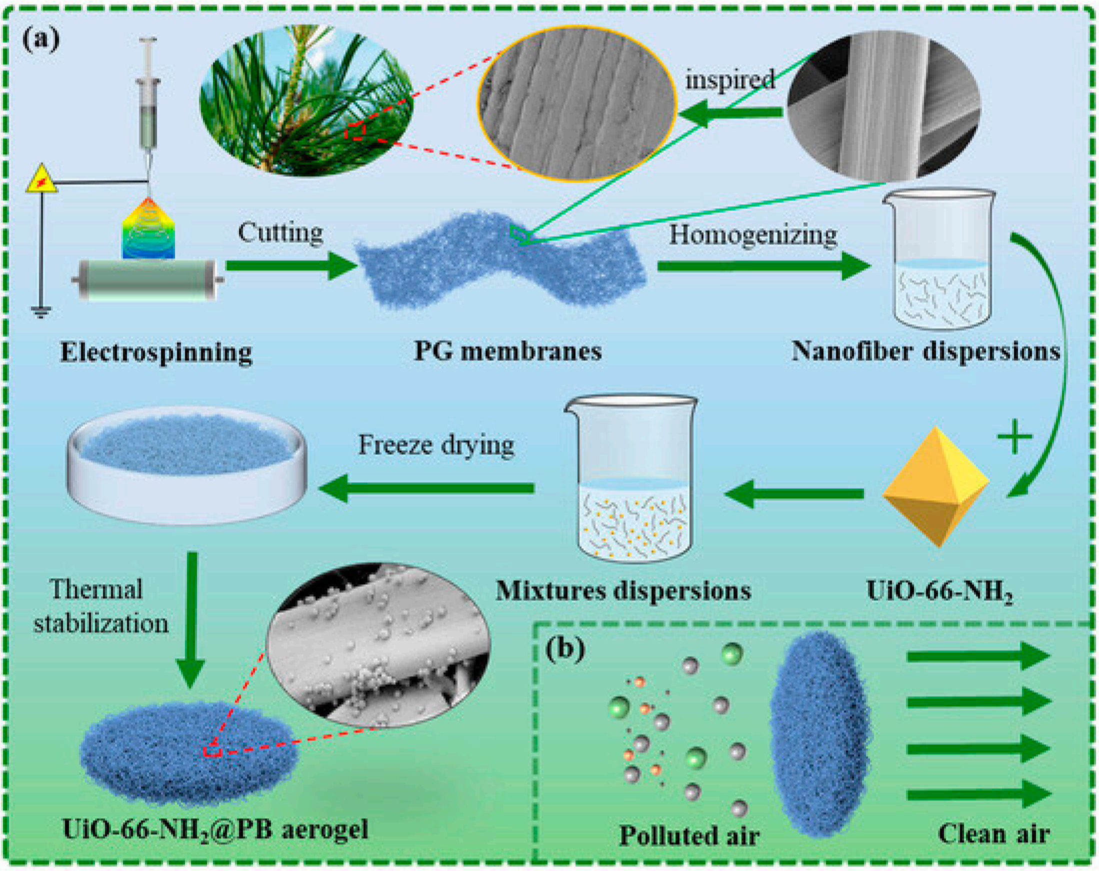

Figure S11. Schematic illustration of the preparation process (**a**) and use for polluted air filtration (**b**) of biomimetic grooved ribbon AG inspired by the structure of *Pinus sylvestris var. mongolica* needles (UPG aerogels). This image has been reproduced by an open access article by Zhao et al. [566] distributed under the terms and conditions of the Creative Commons Attribution (CC BY) license (https://creativecommons.org/licenses/by/4.0/, accessed on 25 August 2025).

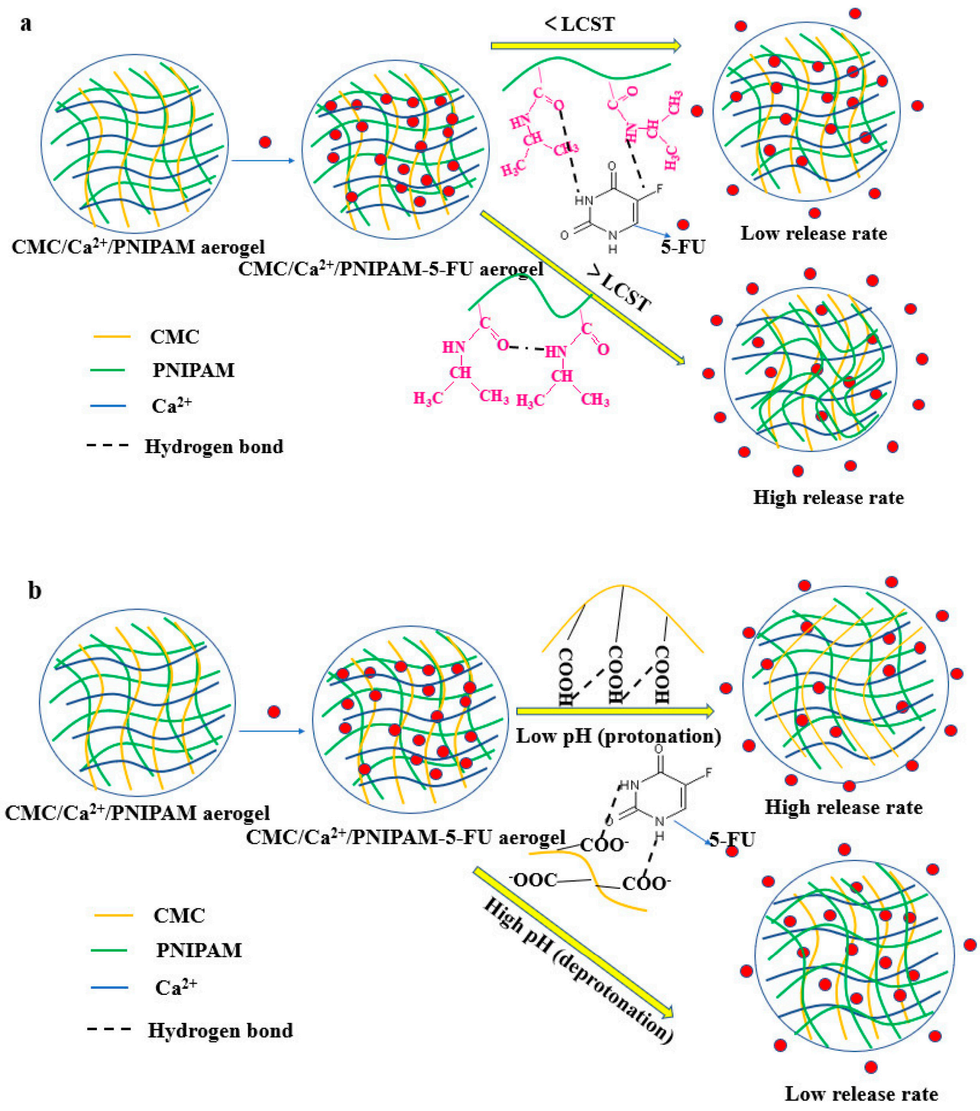

Figure S12. (a) Drug-release process for carboxymethyl cellulose/poly (*N*-isopropyl acrylamide) interpenetrating polymer network aerogels under different temperatures; (b) release process for aerogels under different pH conditions. This image has been reproduced by an open access article by Liu et al. [567] distributed under the terms and conditions of the Creative Commons Attribution (CC BY) license (https://creativecommons.org/licenses/by/4.0/, accessed on 25 August 2025).

Disclaimer/Publisher's Note: The statements, opinions and data contained in all publications are solely those of the individual author(s) and contributor(s) and not of MDPI and/or the editor(s). MDPI and/or the editor(s) disclaim responsibility for any injury to people or property resulting from any ideas, methods, instructions or products referred to in the content.
